# Development and Characterization of a Monoclonal Antibody against Ochratoxin B and Its Application in ELISA

**DOI:** 10.3390/toxins2061582

**Published:** 2010-06-21

**Authors:** Alexandra H. Heussner, Simon Ausländer, Daniel R. Dietrich

**Affiliations:** Human and Environmental Toxicology, University of Konstanz, PO-Box 918, 78457 Konstanz, Germany; Email: alexandra.heussner@uni-konstanz.de (A.H.H.); simon.auslaender@googlemail.com (S.A.)

**Keywords:** mycotoxin, ochratoxin B, monoclonal antibody, ELISA, western analysis

## Abstract

A monoclonal antibody specific to ochratoxin B (OTB) was employed for the development of an indirect competitive OTB-ELISA. The optimized OTB-ELISA resulted in a limit of detection (LOD) for OTB of 3 µg/L (8 nM), a limit of quantification (LOQ) of 3.7 µg/L (10 nM), and a 50% inhibitory concentration (IC_50_) of 150 nM. Due to very low cross-reactivity to OTA (2.7%) and structurally related molecules (0%), this OTB-ELISA was found to be suitable to detect OTB with excellent precision in different matrices, *i.e.*, beer, coffee and wine. Therefore, this OTB-ELISA will allow screening of OTB in food and feed products.

## 1. Introduction

**Figure 1:**
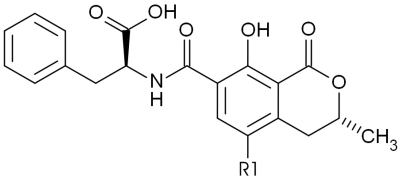
Chemical structures of ochratoxin A and B. Ochratoxin A, R1 = Cl; ochratoxin B, R1 = H.

Ochratoxins are known contaminants of human food and animal feed, frequently detected for example in cereals, coffee, beer and wine [1]. Ochratoxin B (OTB) is the non-chlorinated analog of the naturally mycotoxin ochratoxin A (OTA, Figure 1). Both are secondary metabolites of *Aspergillus* and *Penicillium* species [1], and OTB is therefore frequently found in mold-contaminated products containing OTA, albeit at lower concentrations than OTA [2]. Indeed, a OTB/OTA biosynthesis ratio of approximately 1:5 is reported for *Aspergillus ochraceus* as well as various *Penicillium* species [3] that produce OTA and OTB concentrations in the high ppb range [4]. 

The toxicity of OTA has been intensively studied (for review see [1,5,6,7]) and some of the mechanisms of acute toxicity of OTA are well described [1]. More recently, an epigenetic mode of action underlying chronic OTA toxicity and carcinogenicity in rodents was proposed [5]. Despite the profound species- and sex differences in susceptibility to OTA observed [1] and the known problems in extrapolating human risk and predicting disease from animal data [8], OTA was classified by the IARC as a possible human carcinogen (group 2B) [9]. The latter was based on the available *in vitro* data, *in vivo* animal studies, the human epidemiologic data from the Balkans, and the broad human exposure to OTA, thereby also mandating routine determination of OTA contamination levels in food and animal feed. 

OTB has generally been considered less toxic. However, recent *in vitro* studies have shown that OTB effects are strongly dependent on the model chosen [10,11,12]. Moreover, experimental evidence suggested that OTB has a different mode of action than OTA. Recently, it has been shown in LLC-PK_1_ cells that the cytotoxic effects of OTB were not reversible upon toxin removal, whereas they were reversible with OTA [12]. Similarly, Mally *et al.* [10] demonstrated that the cytotoxic concentrations and effects of OTA and OTB in LLC-PK_1_ cells were comparable. Contrary to OTA, where extreme concentrations *in vitro* were reported to induce micronuclei and DNA migration in the single-cell gel electrophoresis assay, thus suggesting genotoxicity of OTA, OTB had no such effects but rather caused pronounced inhibition of cell division already at much lower concentrations [13]. From the above data, it may be concluded that OTA and OTB have a similar potential to induce cytotoxicity *in vitro*, however large differences are apparent with respect to their potential to induce nephrotoxicity in rats [10]. Indeed, the biotransformation and elimination of OTB appeared more extensive than OTA in rats [10]. Moreover, although OTA is considered to be the major causative factor in Mycotoxic Porcine Nephropathy (MPN) in many European countries [14,15], the role of OTA in a similar human disease (BEN, Balkan Endemic Nephropathy) and in the observed increased incidence of human renal urothelial tumors is still under debate [16,17,18]. The potential role of OTB in both the human and the porcine nephropathy and the human urothelial tumors, has so far largely been ignored and remains to be elucidated.

Based on the above findings, it can be assumed that OTB may pose a risk to humans. Thus, OTB is at least as important as OTA and it may be concluded that routine detection of OTB and OTA in food and beverages would be a prerequisite for improved determination of ochratoxin risk. Indeed, in order to protect consumers from mycotoxin-related risks, the European Union (EU) has defined regulatory limits for OTA, *i.e.*, 10 ppb in dried vine fruits and instant coffee, 5 ppb in cereals and roasted coffee and 2 ppb in wine [19], but has no regulatory limits for OTB. 

Employing high-performance liquid chromatography (HPLC) and liquid chromatography coupled with tandem mass spectrometry (LC/MS/MS), with a limit of quantification (LOQ) of 0.1 µg/kg sample, a recent study demonstrated the co-occurrence of OTA and OTB in commercial foods in Japan [20]. OTA was detectable in 44 of 157 samples, whereas OTB was found in fruit and cacao products containing relatively high levels of OTA. In a similar study [21], OTA and OTB were detected in 143 (21%) and 68 (10%), respectively, in a total of 681 samples. The latter emphasizes that OTB is a contaminant to be seriously considered in the determination of ochratoxin risk. However, although many laboratories already routinely employ HPLC and LC MS/MS technology, more rapid and less cost intensive methodology would be key to a broader, routine and more frequent analysis of ochratoxin B contaminated food and feed.

For above purpose a monoclonal anti-OTB antibody was produced and initially characterized in an earlier study [22]. The overall aim of the current study was to purify and characterize the antibody and to establish and optimize an indirect competitive OTB-ELISA suitable for the high-throughput robust detection of OTB in different food matrices, albeit with the slightly higher LOQ than is typical for HPLC and LC MS/MS technology.

## 2. Materials and Methods

### 2.1. Materials

Stock solutions (1 mM) of OTA and OTB (Sigma-Aldrich) were prepared in PBS and stored in small aliquots at −20 °C.

The OTA-BSA was obtained from Sigma-Aldrich, whereas the OTB-BSA conjugate was synthesized in our laboratory as earlier described [22]. Blocking reagents were purchased from Fluka (Skim milk powder, #70166), Sigma-Aldrich (Ovalbumin, #A5503; Casein, #009000719) and from Roth (BSA, #8076.2). Goat anti-mouse IgG (Fc-Fragment)-HRP (Dianova, #115-035-008) and rabbit anti-mouse IgG-HRP (Sigma, #A9044) were used for ELISA and Western blotting, respectively. ECL (GE Healthcare, #RPN2132) and TMB liquid substrate (Sigma-Aldrich, #T4444) were used as substrates. Unless otherwise stated, all chemicals were purchased from Sigma-Aldrich, Germany, and were of highest purity available.

### 2.2. Production of the OTB Antibody

The monoclonal mouse anti-OTB antibody (OTB mab), IgG1 with κ-chain, was produced using the hybridoma technique. The production and preliminary characterization of this OTB mab was recently described in detail by Heussner *et al.* [22].

### 2.3. Purification and Characterization of the OTB Antibody

For purification, the supernatants from the stable hybridoma cell line 2F1.E10 [22] were collected and stored at −20 °C. Purification was performed using protein G sepharose affinity chromatography according to manufacturer's recommendations (HiTrap, GE Healthcare). Briefly, samples were diluted with binding buffer (20 mM NaH_2_PO_4_, pH 7.0) and applied to the column. After washing, elution was achieved with Glycin-HCl (100 mM, pH 2.7). To avoid globulin damage, pH was titrated to neutral immediately with Tris-HCl (1 M, pH 9.0). 

Eluted fractions were collected and protein content was determined via the Bradford assay calibrated with bovine γ-globulin according to manufacturer’s instructions (Roti®-Quant, Roth, Germany). Additionally, the purity of the protein-containing fractions was assayed via SDS-PAGE under reducing and non-reducing conditions on 12% and 7.5% polyacrylamide gels, respectively. Proteins were stained with silver nitrate [23].

Antibody-containing fractions were pooled and calculated protein content was confirmed via Bradford determination. Purified pooled OTB mab was diluted with glycerol (final concentration of glycerol 50% v/v) and stored in small aliquots at −20 °C.

### 2.4. Characterization of Ochratoxin-BSA Conjugates

Two different ochratoxin-BSA conjugates were used: OTB-BSA (own synthesis, [22]) and OTA-BSA (Sigma-Aldrich). For both, the stability was unknown and confirmation of protein and ochratoxin content were prerequisites for further use in the Western blot analyses (see below). 

Protein concentration was determined via the Bradford micro assay according to the manufacturer’s recommendations (Roti®-Quant, Roth, Germany) using non-linear calibration with BSA. Ochratoxin concentrations were determined by photometric analysis at 360 nm and 380 nm for OTB and OTA, respectively, using non-linear calibration with ochratoxins in Tris buffer. As determination of low ochratoxin concentrations was the ultimate goal, the data were confirmed by additional readings using increasing ochratoxin sample spiking levels (1, 10 and 100 µM).

### 2.5. Detection and Quantification of Ochratoxin-BSA Conjugates via Western Blot Analysis

For semi-quantitative detection, ochratoxin-BSA conjugates were boiled in SDS-PAGE sample buffer (187.5 mM Tris-HCl, pH 8.8, 10% Glycerol, 2% SDS, 20% 2-Mercaptoethanol, 1% Bromophenol blue) and separated on a 10% polyacrylamide gel. Proteins were blotted onto a nitrocellulose membrane (0.2 µm pore size, Roth, Germany), reversibly stained with Ponceau S and blocked with 1% (w/v) BSA in TTBS (100 mM Tris-HCl, pH 7.6, 0.9% (w/v) NaCl, 0.1% (v/v) Tween 20) for 30 min. Thereafter, the OTB mab was used as the primary antibody at a concentration of 10 µg/mL (concentration was optimized in preliminary assays) and incubated for 1 hour at RT. Anti-mouse IgG-HRP (1:80,000, 45 min) and ECL were used for detection. Chemiluminescent signals were detected using a CCD camera system (Fuji LAS-1000). 

For quantification, ochratoxin-conjugates in varying concentrations were applied directly on a nitrocellulose membrane (via dot blotting), followed by immunodetection as described above.

### 2.6. Establishment and Optimization of an Indirect Competitive OTB-ELISA

The initial ELISA was performed using the same parameters as optimized for the use of the crude hybridoma supernatant (see Table 1) [22]. Briefly, 96 well-plates (MaxiSorp, Nunc, Germany) were coated overnight at 4 °C with OTB-BSA conjugate (16 ng/well) in coating buffer (32 mM Na_2_CO_3_, 70 mM NaHCO_3_, pH 9.6). Plates were washed three times with washing buffer and two times with PBS (10 mM sodium phosphate, 150 mM NaCl, pH 7.4) followed by blocking in blocking solution for one hour at RT. Standards, controls or samples (50 µL) were applied and 50 µL OTB mab were added. After one hour incubation, plates were washed again and 100 µL detection antibody (goat anti-mouse IgG (Fc-Fragment)-HRP, 1:5,000) were incubated for one hour. After washing, 50 µL TMB solution were added to each well and incubated 15 min at RT protected from light. The reaction was stopped by addition of 50 µL 2 M sulfuric acid and absorbance was recorded at 450 nm using an ELISA reader (Tecan, Infinite 200).

For optimization of ELISA conditions, grid experiments, allowing for the combination of variations of the different parameters, were performed. Parameters included coating concentrations (8, 16, 32 and 80 ng OTB-BSA per well), OTB mab concentrations (0.007, 0.02, 0.07, 0.2, 0.7 and 1 µg OTB mab/mL), ionic strength (0.1×, 0.5×, 1× and 1.5× PBS), pH (7.0, 7.4 and 8.0), blocking proteins at different concentrations (Casein (0.5, 1, 2%), BSA (1, 2, 3%), Ovalbumin (0.1, 0.2, 0.5%) and skim milk powder (0.1, 0.2, 0.5%)) and incubation conditions (static at 37 °C *vs.* shaking at RT). Four concentrations of OTB and OTA (0.01, 0.1, 1, 10 µM) were used for the latter experiments.

**Table 1:** Comparison of ELISA conditions.

ELISA Step	Before Optimization	After Optimization
Plate coating with OTB-BSA conjugate	16 ng/well	16 ng/well
	coating buffer	coating buffer
	4 °C, overnight	4 °C, overnight
Buffer	PBS (10 mM sodium phosphate, 150 mM NaCl, pH 7.4)	PBS (15 mM sodium phosphate, 225 mM NaCl, pH 7.0)
Washing buffer	PBS with 0.05% (v/v) Tween 20	PBS with 0.05% (v/v) Tween 20
Blocking solution	1% (w/v) casein in PBS	1% (w/v) BSA in PBS
Blocking	RT, 1 h, static	RT, 1 h, static
Primary antibody (mouse anti-OTB IgG)	300 ng IgG/mL blocking solution	70 ng IgG/mL blocking solution
	37 °C, static	RT, shaking
Detection antibody (goat anti-mouse IgG (Fc-fragment)-HRP)	1:5,000 in blocking solution	1:5,000 in blocking solution
	37 °C, static	RT, shaking

### 2.7. Establishment of Competitive Binding Curves of OTB and OTA

Complete competition curves for OTB and OTA were established using, OTA and OTB diluted in PBS (pH 7.0). Concentrations ranged from 10 nM to 6.6 µM and 10 nM to 200 µM for OTB and OTA, respectively. Relative cross-reactivity was determined using Equation 1. 

   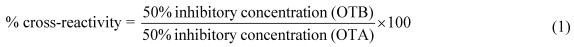   

 The limit of detection (LOD; reagent blank + 3× SD of reagent blank) for OTB was 3 µg/L (8 nM), whereas the limit of quantification (LOQ; reagent blank + 10× SD of reagent blank) was 3.7 µg/L (10 nM). The LOD for OTA was 129 µg/L (320 nM), whereas the LOQ was 174 µg/L (430 nM).

### 2.8. Preparation of Matrix Samples for ELISA

In order to study matrix-associated effects, a very small basket study with beverages was carried out. Beverages randomly chosen in a local supermarket were coffee, beer (German wheat beer) and red wine (Merlot), known to often contain ochratoxins at low levels. Coffee was brewed using a standard filter coffee maker, whereas the beer and wine were used for analysis as taken from the supermarket.

Sample preparation was adapted from the method by Wang and co-workers [24], who optimized sample preparation for the detection of OTA in beverages and foods via ELISA. Briefly, for wine and coffee, 6 mL of sample were mixed with 24 mL chloroform and vigorously shaken for 30 min at RT. Then, 16 mL PBS (pH 9.0) were added and mixed. For phase separation centrifugation (1,560 × *g*, 5 min, RT) was applied and the supernatant (water-phase) was aliquoted into fresh tubes and stored at −20 °C until further use. 

Beer was used without extraction, but was ultrasonically degassed for approximately 15 min and then stored at −20 °C as small aliquots until further use. 

### 2.9. Detection of OTB in Spiked Diluted Matrix Samples via ELISA

Matrix samples (see Section 2.8) were diluted with PBS (pH 7.0) using dilution factors of 2 to 25 for analysis until the absence of matrix-associated quenching effects was observed. Following establishment of optimal dilutions, diluted matrices were spiked with OTB ranging from 10 nM to 6.6 µM.

### 2.10. Calculations and Statistics

Duplicate samples were analyzed in each ELISA plate and run simultaneously with controls for total binding (vehicle, *i.e.*, PBS or diluted sample matrix) and blank (samples without anti-OTB mab). Data were processed first using MS EXCEL 2003. Sample absorbance values were blank-subtracted and then compared to total binding (=100%). Means of the duplicates in each independently run ELISA were used as value of one independent replicate. Means of at least three independent replicates were further processed with GraphPad Prism 4.03 using nonlinear regression (sigmoid dose-response with variable slope) with constraints (0% TOP and 100% BOTTOM) and anchorage points. In addition, the competitor concentration inhibiting 50% binding (IC_50_) was determined. The degree of competition was calculated using Equation 2. 

   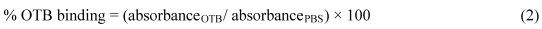   

 Spiked beverage samples were compared to OTB standards in PBS using a t-test with p < 0.05.

## 3. Results

### 3.1. Purification and Characterization of the OTB Antibody

**Figure 2:**
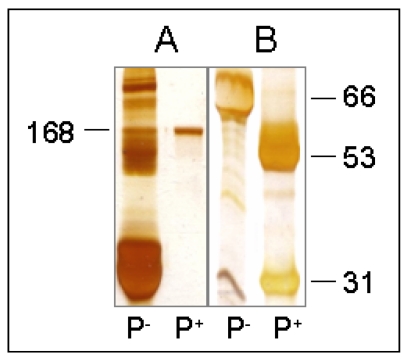
Representative SDS-PAGE results. (A) 7.5% non-reducing gel; (B) 12% reducing gel; P-, before purification; P+, after purification; Numbers refer to estimated molecular weights (kDa).

Antibody purification resulted in a highly purified OTB mab, which was tested via SDS-PAGE with subsequent silver-staining (Figure 2). The gels confirmed the expected distinct banding of immunoglobulin in the absence of any other protein banding. Under reducing conditions, two bands were visible at 53 ± 1 kDa and 31 ± 1 kDa, representing the heavy and light chains, respectively. Under non-reducing conditions, only one protein band at 168 ± 5 kDa was observed. 

### 3.2. Characterization of Ochratoxin-BSA Conjugates

The analysis of OTA-BSA protein concentration confirmed the 5 mg/mL of OTA-BSA as proclaimed by Sigma-Aldrich, while a concentration of 1.3 ± 0.2 µM OTA (n = 3, mean ± SD) fell short of the expected value. For the in-house synthesized OTB-BSA conjugate, a protein concentration of 16.3 ± 1.7 µg/mL (n = 3, mean ± SD) and a OTB concentration of 27.2 ± 1.2 µM (n = 3, mean ± SD) was established. Above ochratoxin-BSA protein concentrations were used for all ensuing calculations, e.g., dot-blots and western blots. 

Above values were also used to calculate the labeling ratios (Ochratoxin: BSA) of 5.3 and 0.36 for OTB-BSA and OTA-BSA, respectively. These values differed from those determined earlier (OTB-BSA, [22]) or provided by the supplier (OTA-BSA, Sigma-Aldrich), suggesting partial loss of ochratoxin-BSA binding during frozen storage.

### 3.4. Detection and Quantification of Ochratoxin-BSA Conjugates via Western Blot Analyses

**Figure 3:**
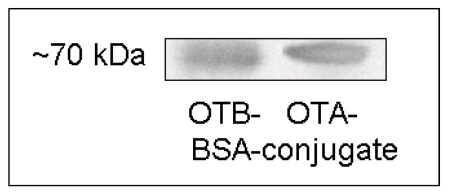
The specificity of OTB- and OTA-protein complex detection by Western blot analysis. A representative Western blot with 1 µg of protein per lane; 10 µg/mL OTB mab; 30 seconds ECL detection.

**Figure 4:**
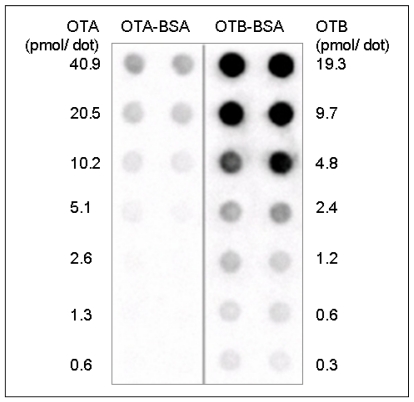
The sensitivity of OTB- and OTA-protein complex detection by Western blot analysis. Representative Dot blot results after 60 seconds ECL detection, 10 µg/mL OTB mab, 30 µL sample/dot.

Western blot analysis enabled the control of the ELISA components, thus confirming the previously described method [22]. In addition, Western blot analysis tested and confirmed the potential usability of the OTB mab for the analysis of OTB-protein complexes. Moreover, Western blot analysis demonstrated that the OTB mab is capable of detecting OTB- and OTA-protein complexes with similar specificity (Figure 3), albeit with different sensitivity (Figure 4). 

Whether this approach is applicable to the detection of non-covalently bound, but chemically matrix-linked OTB (e.g., in immunocytochemistry) will be established in future experiments.

### 3.5. Establishment and Optimization of an Indirect Competitive OTB-ELISA

Based on the variant combinations of ELISA parameters used in the grid experiments, the most ideal ELISA conditions were identified (Table 1). The comparison of the conditions employed in the initial ELISA with those of the optimized ELISA demonstrated that not only a simpler but also more convenient (RT *vs.* 37 °C incubation) ELISA was optimal. The most effective modification was the choice of protein for blocking. The use of BSA, rather than casein, ovalbumin or skim milk resulted in highest sensitivity towards OTB and lowest cross-reactivity towards OTA. This may due to the high affinity of OTA to BSA. 

**Figure 5:**
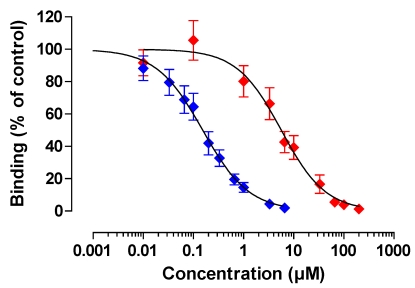
Competitive binding curves of OTB and OTA. Blue symbols, OTB (n = 5), red symbols, OTA (n = 6); Data are means ± SEM.

These optimized conditions were used to investigate competitive effects of OTB and structurally related molecules e.g., OTA, coumarin and phenylalanine. No cross-reactivity to other structurally related molecules (coumarin and phenylalanine) was observed (data not shown), confirming earlier findings [22]. Detection could be achieved within a range of 0.01 to 10 µM OTB (Figure 5). The OTB concentration causing 50% inhibition (IC_50_) was 0.15 µM (95% CI: 0.12–0.19, n = 5), suggestive of a very sensitive assay. The limit of detection (LOD; reagent blank + 3× SD of reagent blank) for OTB was 3 µg/L (8 nM), whereas the limit of quantification (LOQ; reagent blank + 10× SD of reagent blank) was 3.7 µg/L (10 nM). 

OTA was detected with an IC_50_ of 5.65 µM (95% CI: 4.18-7.65, n = 6). Based on the IC_50_’s determined, the cross-reactivity of OTA with the OTB mab was calculated to be very low (2.7%). The LOD for OTA was 129 µg/L (320 nM), whereas the LOQ was 174 µg/L (430 nM).

### 3.6. Matrix Interference and Its Removal

Reduction of matrix interference is frequently achieved in ELISA by simple dilution with assay buffer, especially when a highly sensitive assay is available. Samples containing high pigment, flavors, tannin or lipids generally interfere with the color development in ELISA. Therefore, chloroform extraction with subsequent alkaline buffer dilution, where the interfering substances are retained in the chloroform layer and toxins (in this case ochratoxins) are easily dissolved in alkaline solution, was chosen. 

**Figure 6:**
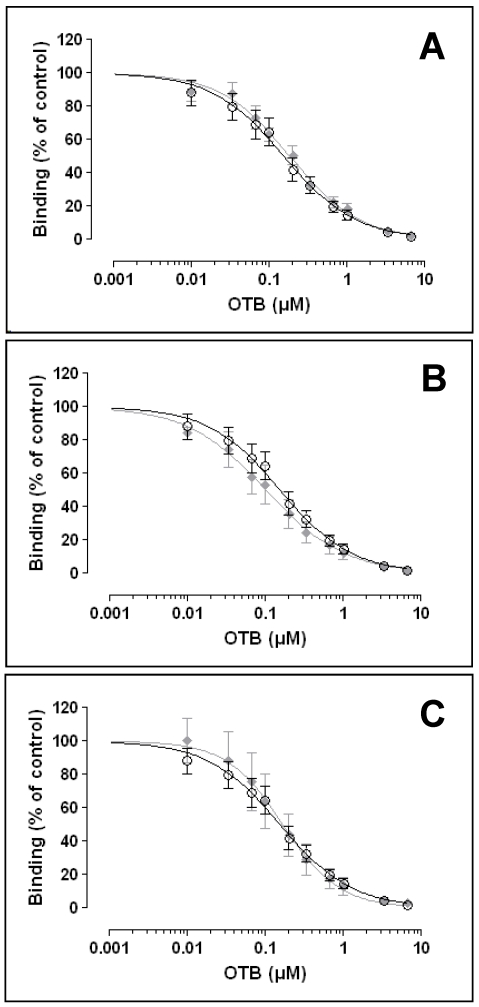
Competitive binding curves of OTB in different matrices. Black symbols, pure OTB standards (n = 5), grey symbols, OTB spiked into matrices; (A) red wine (n = 3); (B) coffee (n = 4); (C) beer (n = 3); Data are means ± SEM.

In a first set of analyses, prepared matrix samples were diluted with PBS pH 7.0 using dilution factors of 2 to 25 for analysis, *i.e.*, until no quenching effects of the matrices were detectable. Based on the latter results, no quenching effects were detected (Figure 6) at dilutions of 1:2, 1:10 and 1:20 for coffee, beer and wine, respectively.

### 3.7. Detection of OTB in Various Matrices

Ten different concentrations of OTB were added (spiking experiment) to diluted matrix samples using the optimized dilutions of the respective sample (matrix) type (see above). The resulting spiked matrix samples were analyzed using the optimized OTB ELISA. The resulting competition curves were compared to that established with pure OTB in PBS (Figure 6). No significantly differences in the curves (form, slope, range) could be detected for all three matrices (coffee, beer, wine), when compared to the standard OTB curve. This t suggests that the optimized OTB ELISA provides for a robust and sensitive OTB detection in these matrices.

## 4. Discussion

Despite that the role of OTA in a human disease (BEN, Balkan Endemic Nephropathy) and in the observed increased incidence of human renal urothelial tumors is still under debate [16], the European Union (EU) has defined regulatory limits for OTA, *i.e.*, 10 ppb in dried vine fruits and instant coffee, 5 ppb in cereals and roasted coffee and 2 ppb in wine, whereas no limits were set for beer [19], in order to protect consumers from mycotoxin-related risks. Similarly, although the potential role of OTB in both, the human and the porcine nephropathy and the human urothelial tumors, has so far largely been ignored and remains to be elucidated, the toxicological database available to date would suggest that OTB should be considered as seriously as OTA as causative toxins in the observed increased incidence of human renal urothelial tumors. Moreover, OTB was demonstrated to be co-produced with OTA and is therefore frequently found in OTA-contaminated products [2,20,21]. Consequently, single or combined exposures to OTA and OTB could be causative for the observed human diseases and as such should be monitored routinely in order to protect the consumer. Although HPLC and LC-MS/MS are established technologies available for most product testing laboratories, the running cost and skilled personnel required can be prohibitive for routine high-throughput analyses of OTA and OTB in food and feed products including beverages e.g., beer, wine and liquors. Here we present an indirect competitive OTB-ELISA with a low detection limit and a broad quantification range. This OTB-ELISA could provide an alternative approach with high-throughput capacity. Indeed, using the limit levels set for OTA by the EU and assuming that similar limits would be applied to OTB, the LOQ of 3.7 ppb that was established for the indirect competitive OTB-ELISA would suffice for routine quantification of OTB contamination in dried vine fruits, instant coffee, cereals and roasted coffee. As the OTB mab shows very high sensitivity (pg range for OTB) and specificity for OTB-BSA, very low cross-reactivity to OTA-BSA, and no cross-reactivity to either coumarin or phenylalanine, specific identification of the contaminant (OTA or OTB) is possible. 

Due to the high specificity of the OTB mab, as demonstrated in Western and dot blotting applications, this antibody could potentially find use in other applications, e.g., immunohistochemistry and immunocytochemistry, and thus help in elucidating the causality of OTB exposure and etiology or progression of Balkan Endemic Nephropathy and the observed increased incidence of human renal urothelial tumors.

In summary, the described OTB mab is suitable for use in ELISA and Western analysis with high specificity and sensitivity for OTB. Moreover, the indirect competitive OTB-ELISA, which was optimized in the present study, was shown to be a sensitive and specific tool to detect OTB in various matrix samples with only minor sample pretreatment which could be applied as a rapid and low cost high-throughput technology for future food sample analysis.
